# Targeting NG2 relieves the resistance of BRAF-mutant thyroid cancer cells to BRAF inhibitors

**DOI:** 10.1007/s00018-024-05280-6

**Published:** 2024-05-25

**Authors:** Fang Sui, Guanjie Wang, Juan Liu, Mengmeng Yuan, Pu Chen, Yao Yao, Shaoqiang Zhang, Meiju Ji, Peng Hou

**Affiliations:** 1 Department of Endocrinology, The First Affiliated Hospital of Xi’an Jiaotong University, Xi’an, Shaanxi Province 710061 P.R. China; 2 Department of Otorhinolaryngology-Head and Neck Surgery, The First Affiliated Hospital of Xi’an Jiaotong University, Xi’an, Shaanxi Province 710061 P.R. China; 3 Center for Translational Medicine, The First Affiliated Hospital of Xi’an Jiaotong University, Xi’an, Shaanxi Province 710061 P.R. China; 4 Key Laboratory for Tumor Precision Medicine of Shaanxi Province, The First Affiliated Hospital of Xi’an Jiaotong University, Xi’an, 710061 P.R. China

**Keywords:** *BRAF* mutations, NG2, Thyroid cancer, Receptor tyrosine kinase (RTK), Feedback activation

## Abstract

*BRAF*^*V600E*^ represents a constitutively active onco-kinase and stands as the most prevalent genetic alteration in thyroid cancer. However, the clinical efficacy of small-molecule inhibitors targeting *BRAF*^*V600E*^ is often limited by acquired resistance. Here, we find that nerve/glial antigen 2 (NG2), also known as chondroitin sulfate proteoglycan 4 (CSPG4), is up-regulated in thyroid cancers, and its expression is increased with tumor progression in a *BRAF*^*V600E*^-driven thyroid cancer mouse model. Functional studies show that NG2 knockout almost does not affect tumor growth, but significantly improves the response of BRAF-mutant thyroid cancer cells to BRAF inhibitor PLX4720. Mechanistically, the blockade of ERK-dependent feedback by BRAF inhibitor can activate receptor tyrosine kinase (RTK) signaling, causing the resistance to this inhibitor. NG2 knockout attenuates the PLX4720-mediated feedback activation of several RTKs, improving the sensitivity of BRAF-mutant thyroid cancer cells to this inhibitor. Based on this finding, we propose and demonstrate an alternative strategy for targeting NG2 to effectively treat BRAF-mutant thyroid cancers by combining multiple kinase inhibitor (MKI) Sorafenib or Lenvatinib with PLX4720. Thus, this study uncovers a new mechanism in which NG2 contributes to the resistance of BRAF-mutant thyroid cancer cells to BRAF inhibitor, and provides a promising therapeutic option for BRAF-mutant thyroid cancers.

**Supplementary Information:**

The online version contains supplementary material available at 10.1007/s00018-024-05280-6.

## Introduction

*BRAF*^*V600E*^ mutation is the most common genetic event in thyroid cancers [1–3]. Thyroid cancers harboring this mutation have been reported to exhibit more aggressive tumor behaviors, including extrathyroidal extension, lymph node metastases, loss of iodine avidity, and higher risks of recurrence and disease-related death [4–6]. Thus, the use of small molecular inhibitors targeting mutant BRAF and its downstream effector MEK has become a major therapeutic strategy for BRAF-mutant thyroid cancers [4–6].

The initial approved selective inhibitor targeting mutant BRAF is vemurafenib (PLX4032), showcasing promising early therapeutic benefits [7]. Despite the improved survival observed in patients with BRAF-mutant metastatic melanomas by the use of BRAF inhibitors [7], their efficacy is limited in individuals with thyroid cancer [8, 9]. This limitation can be attributed to the rebound effect in extracellular signal-regulated kinase (ERK) activity, which is mediated by heightened HER2/HER3 signaling [9]. Lapatinib as a small molecule dual tyrosine kinase inhibitor of EGFR and HER-2 has been demonstrated the ability to prevent the rebound of the ERK signaling and sensitize BRAF-mutant thyroid cancer cells to RAF or MEK inhibitors [10]. Similarly, a recent study showed that BRAF inhibitors vemurafenib and dabrafenib exhibited moderate antitumor effect and stronger efficacy when combined with receptor tyrosine kinase (RTK) inhibitors in 9 patient-derived papillary thyroid cancer (PTC) organoids [11]. These observations suggest that a combination of BRAF inhibitor with RTK inhibitors holds the potential to improve the therapeutic efficacy for BRAF-mutant thyroid cancers. However, the mechanism underlying the resistance of BRAF-mutant thyroid cancers to its specific inhibitors is far more complex than we thought.

Cell surface proteoglycans are known to be important regulators for the activity of RTK signaling, including nerve/glial antigen 2 (NG2), which is also known as chondroitin sulfate proteoglycan 4 (CSPG4), and heparan sulfate proteoglycans (HSPGs) [12, 13]. Among them, NG2 as a co-receptor of some RTKs has been evidenced to enhance growth factor receptor-regulated signaling pathways including sustained activation of ERK signaling [14]. These observations raise two interesting questions: (1) whether NG2 may be potential therapeutic target for thyroid cancers, especially particularly those with BRAF mutations; (2) whether NG2 is involved in the resistance of BRAF-mutant thyroid cancers to BRAF inhibitors.

In the present study, we found that high expression of NG2 was correlated with the progression of thyroid cancer. In addition, we demonstrated that, although NG2 did not affect tumor growth in vitro and in vivo, it decreased the sensitivity of BRAF-mutant thyroid cancer cells to BRAF inhibitor by regulating the activities of several major RTKs.

## Results

### NG2 is highly expressed in thyroid cancer and associated with tumor progression

**Fig. 1:**
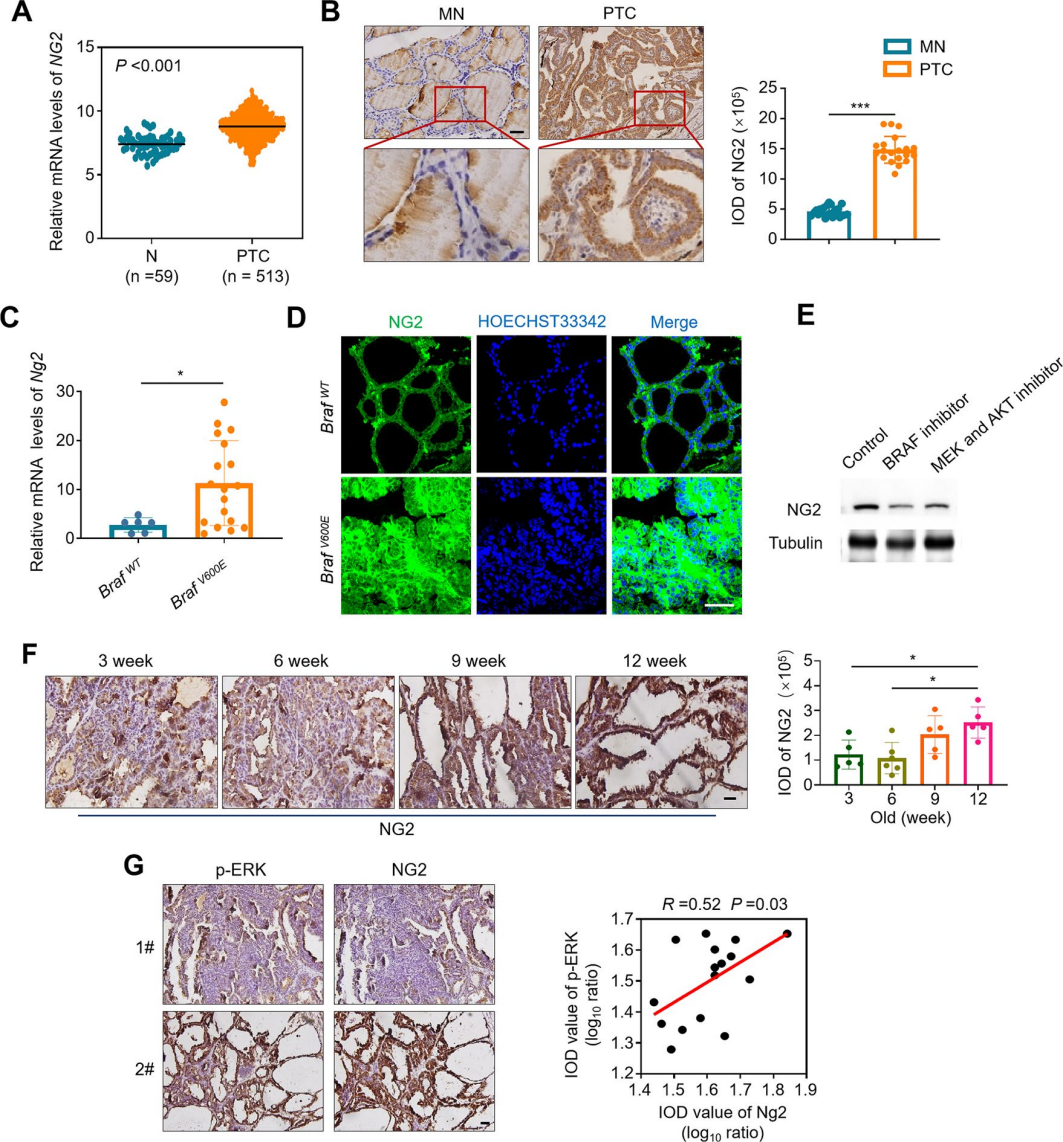
NG2 is up-regulated in thyroid cancers and correlated with tumor progression. (**A**) The mRNA expression of NG2 in PTCs and adjacent normal thyroid tissues (N) (the data from TCGA database). (**B**) IHC staining showing NG2 expression in 20 pairs of PTCs and their matched non-cancerous tissues (MNs) (left panels). Representative IHC images were shown in the left panel. Quantitative illustration of NG2 proteins was shown in the right panel. Scale bars, 50 μm. (**C**) qRT-PCR assay showing the relative mRNA expression of *Ng2* in thyroid tissues of Braf^V600E^-driven thyroid cancer mouse model (*Braf*^*V600E*^) and controls (*Braf*^*WT*^). *18 S* rRNA was used as a reference gene. (**D**) Immunofluorescence assay showing NG2 expression in thyroid tissues of Braf^V600E^-driven thyroid cancer mouse model and controls (*Braf*^*WT*^). Scale bars, 50 μm. (**E**) Western blot assay showed the expression of NG2 upon BRAF inhibitors (1 µM PLX4720) or MEK and AKT inhibitor (1 µM GSK1120212 and 5 µM MK-2206) treatment. (**F**) Immunohistochemistry (IHC) staining showing NG2 expression in thyroid tissues of *Braf*^*V600E*^ mice aged 3 weeks, 6 weeks, 9 weeks and 12 weeks. Left panels show the representative IHC images, and quantitative illustration of NG2 proteins with IOD value was shown in the right panel. (**G**) Left panels show the representative IHC images of p-ERK and NG2 in thyroid tissues of *Braf*^*V600E*^ mice, and right panel shows the correlation analysis between the expression of NG2 and the levels of p-ERK. Data were presented as mean ± SD. IOD, integrated optical density. *, *P* < 0.05; ***, *P* < 0.001

We first analyzed the expression of NG2 in PTCs and non-cancerous thyroid tissues (control subjects) using the Cancer Genome Atlas (TCGA) database. The results showed that NG2 was up-regulated in PTCs compared with control subjects (Fig. 1A). Also, we further validated elevated expression of NG2 in PTCs compared with their matched non-cancerous thyroid tissues by immunohistochemistry (IHC) staining (Fig. 1B). In addition, we established BRAF^V600E^-driven transgenic mouse model of thyroid cancer (*TPO-Cre/LSL-Braf*^*CA*^) as described previously [15], and demonstrated that Ng2 was significantly increased in tumor tissues from BRAF-mutant mice (*BRAF*^*V600E*^) compared with wild-type ones (*BRAF*^*WT*^) by qRT-PCR and immunofluorescence (IF) assays (Fig. 1C, D). Further Western blot assay shows that BRAF inhibitors could down-regulate NG2, and the inhibition of its downstream (MEK and AKT) could also inhibit NG2. Therefore, the activation of BRAF and its downstream may lead to the abnormal expression of NG2 (Fig. 1E).

By examining the expression levels of Ng2 in tumor tissues of BRAF-mutant mice aged 3 weeks to 12 weeks, we found that its expression increased with tumor progression (Fig. 1F). Besides, we analyzed the expression of NG2 in a panel of PTC samples, and found a positive correlation between the expression of NG2 and the levels of phosphorylated ERK (p-ERK) in these tissues (Fig. 1G). These data suggest that there is a link between NG2 and the activation of ERK signaling, which was consistent with previous studies [14, 16].

### NG2 knockout does not affect thyroid cancer growth in vitro and in vivo

**Fig. 2:**
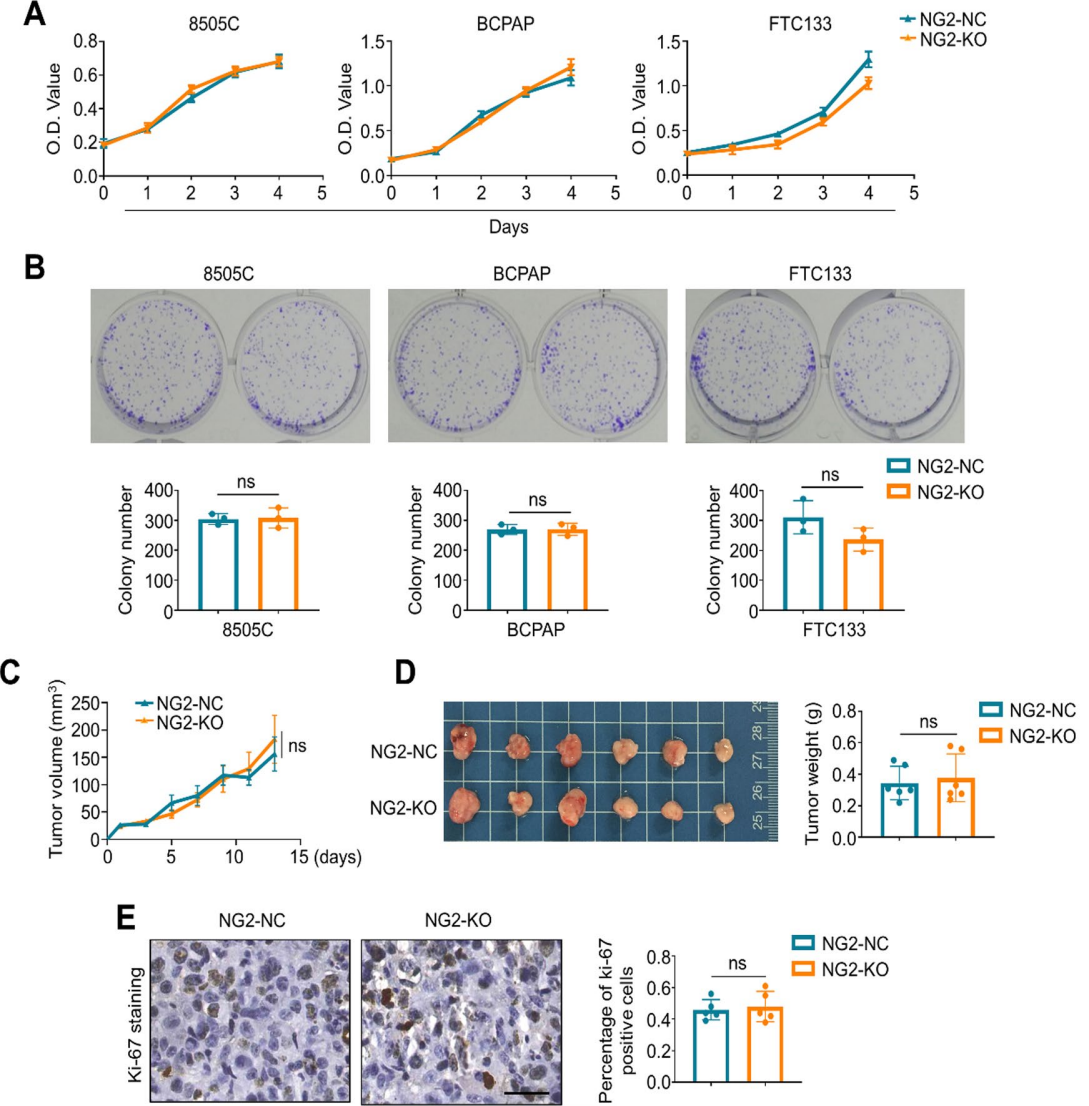
*NG2* knockout has little effect on thyroid cancer growth. (**A**) The effect of *NG2* knockout on the proliferation in 8505 C, BCPAP and FTC133 cells was monitored by MTT assay. (**B**) The effect of *NG*2 knockout on colony formation abilities of 8505 C, BCPAP and FTC133 cells. Upper panels show the representative images of colony formation, and lower panels show the quantitative analysis of colonies. (**C**) Growth curves of NG2-knockout 8505 C cell-derived xenograft tumors (NG2-KO) or control tumors (NG2-NC) in nude mice (*n* = 6/group). (**D**) Left and right panels show the photographs and mean weight of dissected NG2-knockout xenograft tumors and control tumors. (**E**) IHC staining shows the levels of Ki-67 in the indicated xenograft tumors. The representative IHC images were presented on the left panels, and quantitative analysis was shown on the right panels. Scale bars, 25 μm. Data were presented as mean ± SD. ns, no significance

Next, we performed a series of functional studies to determine the biological function of NG2 in thyroid cancer. First, we knocked out NG2 in 8505 C, BCPAP and FTC133 cells (Supplementary Fig. 1), and found that NG2 knockout virtually did not affect the proliferation and colony formation abilities of these cells compared with the control (Fig. 2A, B). Moreover, we also established the xenograft tumor models in nude mice using NG2-knockout 8505 C cells and control cells, and evaluated their tumorigenicity. The results showed that NG2 knockout did not affect tumor growth (Fig. 2C) and tumor weight (Fig. 2D) compared with the control. This was also supported by the results of Ki-67 staining showing that the percentage of Ki-67 positive cells was virtually unchanged upon NG2 knockout (Fig. 2E).

**Fig. 3:**
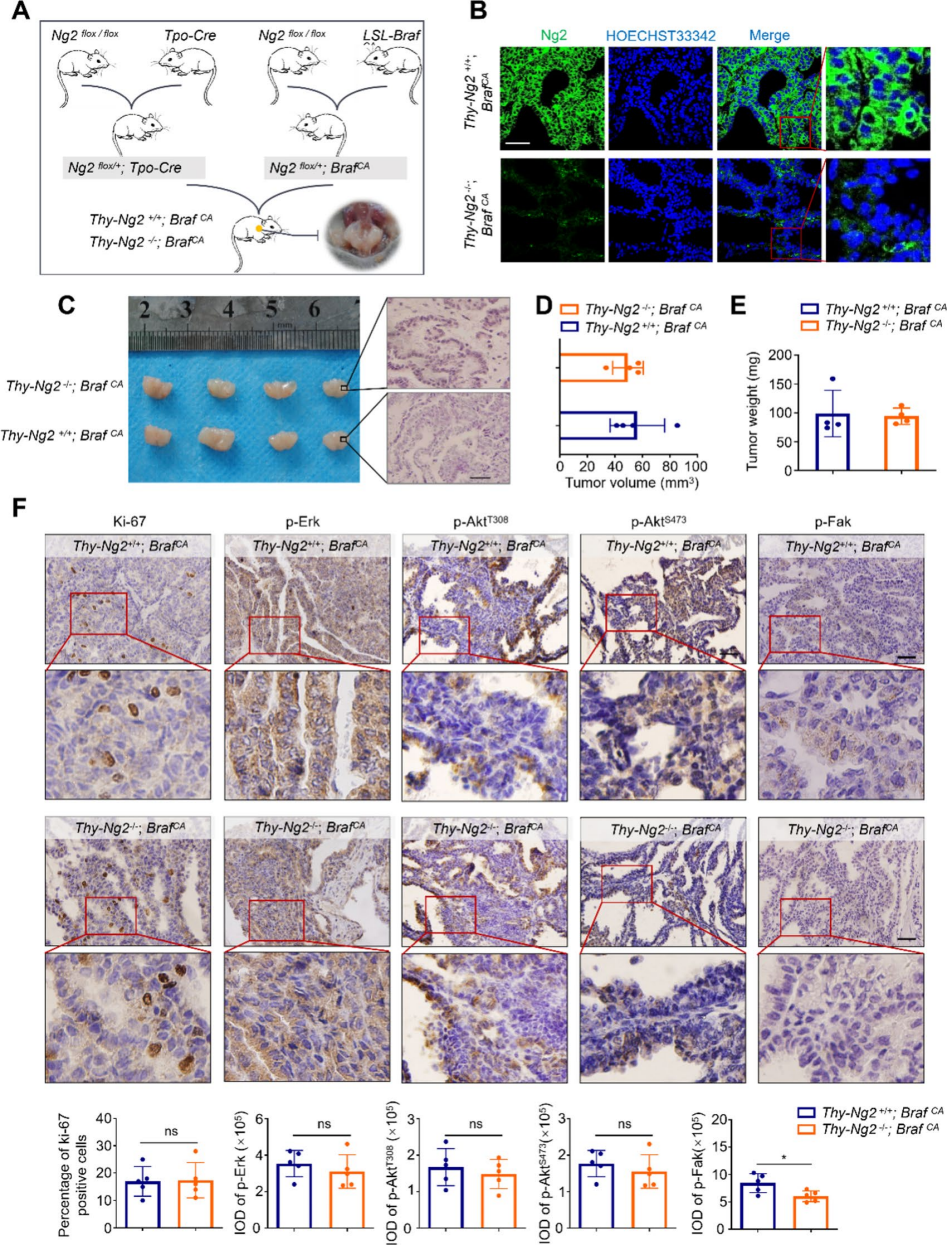
Ng2 knockout has little effect on tumor growth in genetically engineered mice. (**A**) Ng2-knockout Braf^V600E^-driven thyroid cancer mouse model (*Thy-Ng2*^−/−^; *Braf*^*CA*^) and control model (*Thy-Ng2*^+/+^; *Braf*^*CA*^) were constructed by crossing *Ng2*^*flox/flox*^ mice, peroxidase-Cre (*Tpo-Cre*) mice and *LSL-Braf*^*CA*^ mice. (**B**) Immunofluorescence assay was performed to evaluate Ng2 expression in thyroid tissues of *Thy-Ng2*^−/−^; *Braf*^*CA*^ and *Thy-Ng2*^+/+^; *Braf*^*CA*^ mice. (**C**) The photographs (left panel) and representative H&E staining (right panels) of thyroid tumors from *Thy-Ng2*^−/−^; *Braf*^*CA*^ and *Thy-Ng2*^+/+^; *Braf*^*CA*^ mice (*n* = 4/group). Comparison of tumor volume (**D**) and tumor weight (**E**) between *Thy-Ng2*^−/−^; *Braf*^*CA*^ and *Thy-Ng2*^+/+^; *Braf*^*CA*^ mice. (**F**) Immunohistochemical staining was performed to detect the levels of Ki-67, p-ERK, p-AKT^T308^, p-AKT^S473^ and p-FAK in thyroid tumors of the indicated genetically engineered mice. Upper panels show the representative IHC images (red boxes represent amplified images), and lower panels show the statistic results. IOD, integrated optical density. Scale bars, 50 μm. Data were presented as mean ± SD. *, *P* < 0.05; ns, no significance

To further validate the above findings, we generated NG2-knockout coupled with *Braf*^*V600E*^-driven animal model of thyroid cancer (*Thy-Ng2*^*−/−*^; *Braf*^*CA*^) by crossing *LSL-BRAF*^*CA*^, *Ng2*^*flox/flox*^ and peroxidase-Cre (Tpo-Cre) mice, and their wild-type littermates were used as controls (*Thy-Ng2*^*+/+*^; *Braf*^*CA*^) (Fig. 3A). Next, we performed immunofluorescence assays in thyroid tissues of *Thy-Ng2*^*−/−*^; *Braf*^*CA*^ and *Thy-Ng2*^*+/+*^; *Braf*^*CA*^ mice, and found that Ng2 was significantly down-regulated in the former compared with the latter (Fig. 3B), indicating that Ng2 was successfully knocked out in thyroid glands of *Thy-Ng2*^*−/−*^; *Braf*^*CA*^ mice. We sacrificed mice at 12-week-old, collected thyroid tumors and measured their volume and weight. However, we failed to find significant differences in tumor volume and tumor weight between *Thy-Ng2*^*−/−*^; *Braf*^*CA*^ and *Thy-Ng2*^*+/+*^; *Braf*^*CA*^ mice (Fig. 3C, D,E). Some of tumor samples were embedded in paraffin and sectioned for IHC staining. As shown in (Fig. 3F), the levels of Ki-67, p-Erk, phosphorylated Akt ^T308^ (p-AKT^T308^), phosphorylated Akt ^S473^ (p-Akt^S473^) were obviously unchanged in tumor tissues between Thy-Ng2^−/−^; *Braf*^*CA*^ and *Thy-Ng2*^*+/+*^; *Braf*^*CA*^ mice; however, the levels of phosphorylated Fak (p-FAK) were significantly decreased in tumor tissues of *Thy-Ng2*^*−/−*^; Braf^CA^ mice compared with *Thy-Ng2*^*+/+*^; *Braf*^*CA*^ mice, suggesting that NG2 may contribute to the metastatic phenotypes of thyroid cancer cells.

### NG2 knockout improves the response of BRAF-mutant thyroid cancer cells to BRAF inhibitor

**Fig. 4:**
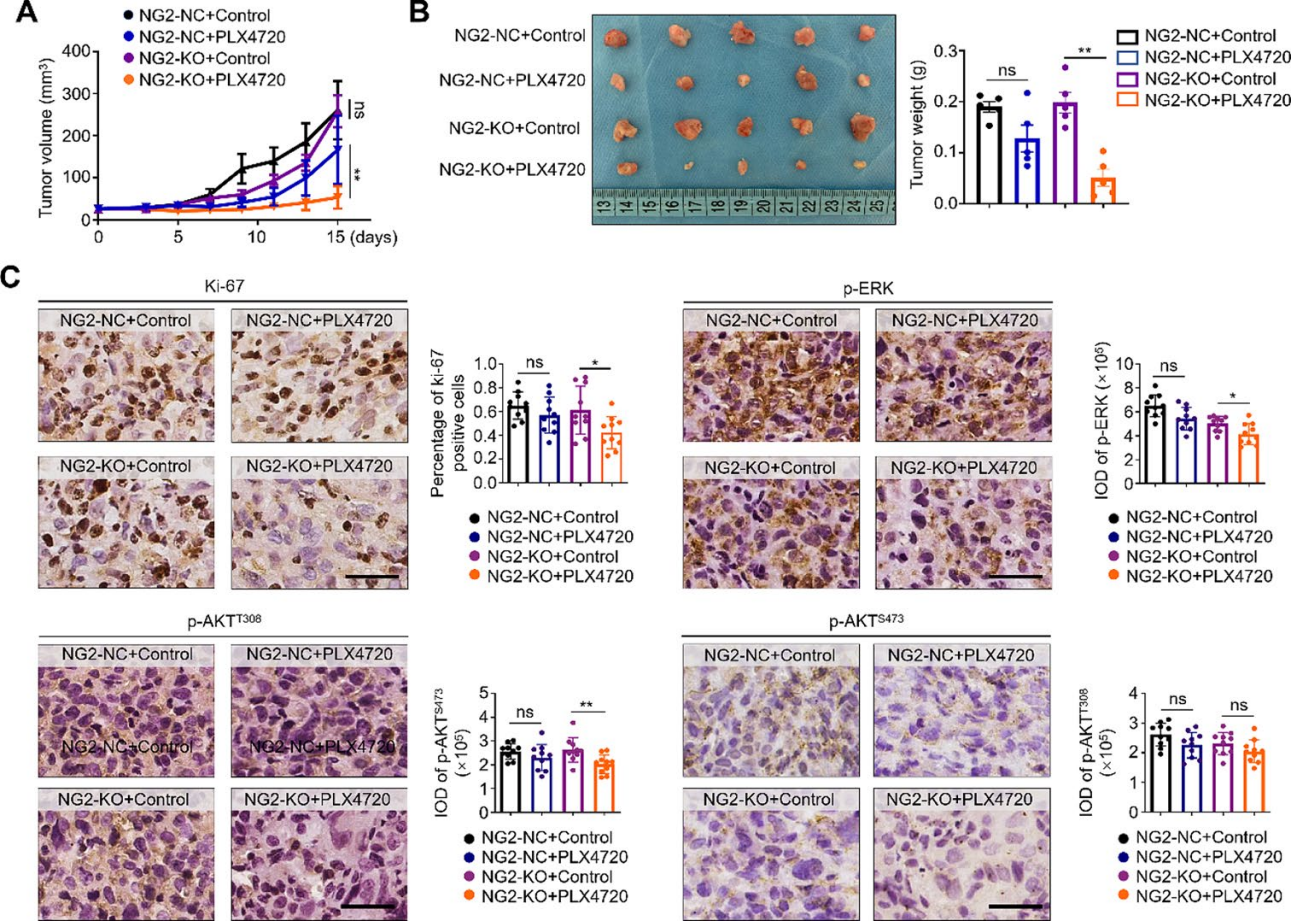
*NG2* knockout increases the sensitivity of BRAF-mutant thyroid cancer cells to BRAF inhibitor. (**A**) Growth curves of xenograft tumors in the indicated groups (*n* = 5/group). (**B**) Left panel shows the images of dissected tumors from the indicated groups, and right panel shows the statistical analysis of tumor weight. (**C**) IHC staining was performed to evaluate the levels of Ki-67, phospho-ERK (p-ERK), phospho-AKT^Thr308^ (p-AKT^Thr308^) and phospho-AKT^Ser473^ (p-AKT^Ser473^) in tumor tissues from the indicated groups. Left panels show the representative IHC images, and right panels show the statistic results. IOD, integrated optical density. Scale bars, 50 μm. NG2-KO, NG2 knockout; NG2-NC, control. Data were presented as mean ± SD. *, *P* < 0.05; **; *P* < 0.01; ns, no significance

Considering that NG2 can maintain the activities of receptor tyrosine kinases (RTKs) and their downstream signaling pathways, we next investigated whether NG2 knockout could reverse the feedback activation of RTKs-mediated ERK and AKT signaling pathways upon BRAF inhibitor treatment. To confirm this, we first constructed xenograft tumor models by subcutaneously injecting NG2-knockout 8505 C cells and control cells. When tumors became palpable, mice were randomly divided into four groups, and received DMSO (as controls) and BRAF inhibitor PLX4720 treatment (20 mg/kg), respectively. During this process, we recorded tumor growth curves and then sacrificed mice after a 15-day treatment. As shown in (Fig. 4A), PLX4720-treated control tumors (NG2-NC) grew slowly compared with DMSO-treated ones, but did not reach a statistical difference. However, PLX4720 treatment significantly delayed the growth of NG2-knockout tumors (NG2-KO) compared with DMSO treatment. As supported, our data showed that, compared with DMSO treatment, PLX4720 treatment significantly decreased tumor weight in NG2-knockout tumors, but not in control tumors (Fig. 4B). These findings indicate that NG2 knockout enhances the sensitivity of BRAF-mutant thyroid cancer cells to BRAF inhibitor. As supported, the results of IHC staining showed that PLX4720 treatment caused a significant decrease in the number of Ki-67-positive cells and the levels of p-ERK and p-AKT^T308^ in NG2-knockout tumors, but not in control tumors (Fig. 4C).

**Fig. 5:**
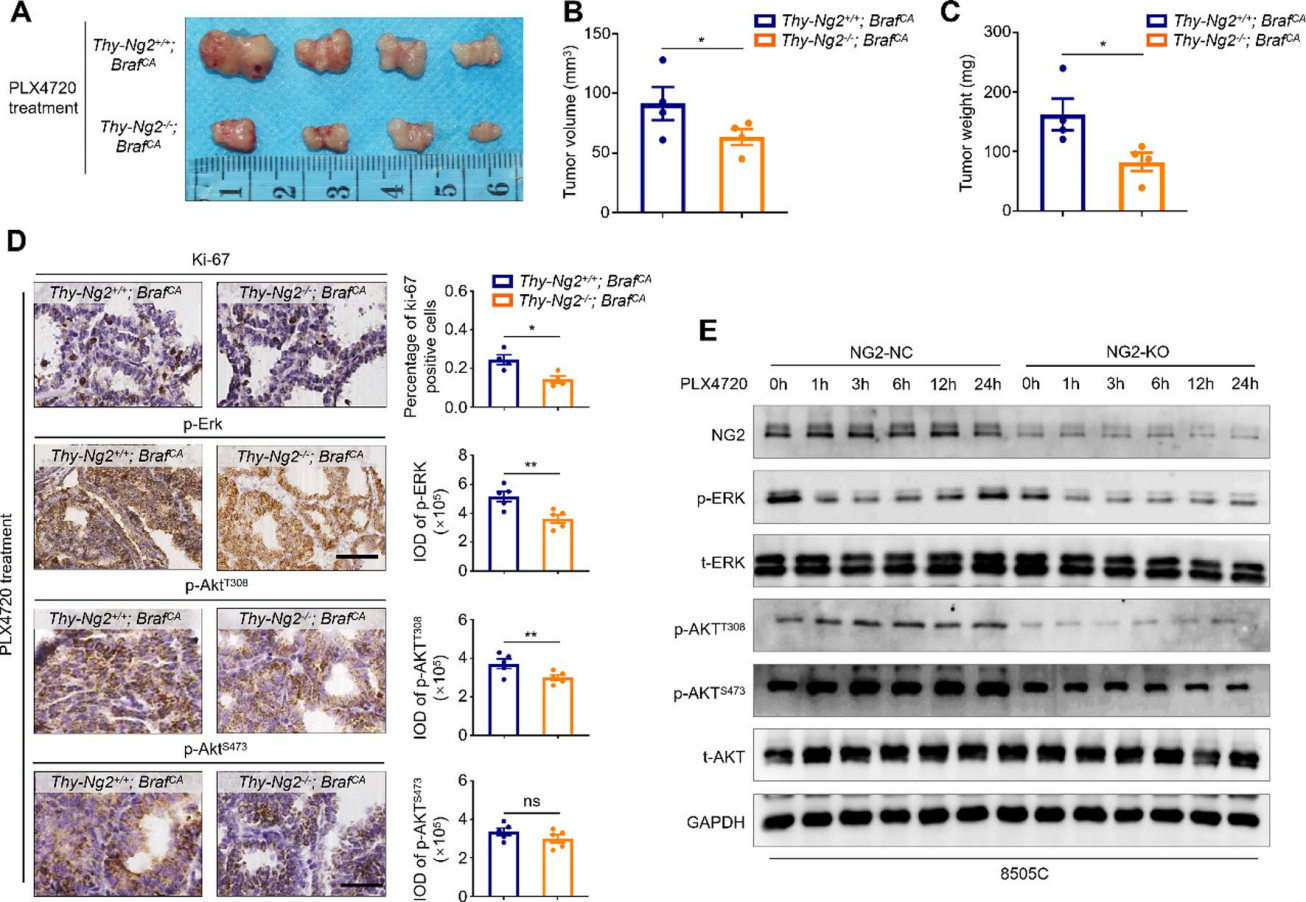
Ng2 knockout improves the response of BRAF-mutant thyroid tumors to BRAF inhibitor in genetically engineered mice. (**A**) Tumor photographs of *Thy-Ng2*^−/−^; *Braf*^*CA*^ and *Thy-Ng2*^+/+^; *Braf*^*CA*^ mice treated with BRAF inhibitor PLX4720. (**B**) Comparison of tumor volume (**B**) and tumor weight (**C**) between *Thy-Ng2*^−/−^; *Braf*^*CA*^ and *Thy-Ng2*^+/+^; *Braf*^*CA*^ mice upon PLX4720 treatment. (**D**) IHC staining was used to detect the levels of Ki-67, p-Erk, phospho-Akt^Thr308^ (p-Akt^Thr308^) and phospho-Akt^Ser473^ (p-Akt^Ser473^) in thyroid tissues of *Thy-Ng2*^−/−^; *Braf*^*CA*^ and *Thy-Ng2*^+/+^; *Braf*^*CA*^ mice upon PLX4720 treatment. Left panels show the representative IHC images, and right panels show the statistic results. Scale bars, 50 μm. (**E**) Western blot analysis was performed to evaluate the effect of NG2 knockout on the levels of p-ERK, p-AKT^Thr308^ and p-AKT^Ser473^ in 8505 C cells upon PLX4720 treatment. GAPDH was used as a loading control. Data were presented as mean ± SD. IOD, integrated optical density; *, *P* < 0.05; **, *P* < 0.01; ns, no significance

To further validate the above conclusions, we treated *Thy-Ng2*^*−/−*^; *Braf*^*CA*^ and *Thy-Ng2*^*+/+*^; *Braf*^*CA*^ mice with PLX4720 (20 mg/kg) for 2 weeks, and measured their tumor volume and weight. The result showed that PLX4720 treatment significantly decreased tumor volume and weight in *Thy-Ng2*^*−/−*^; *Braf*^*CA*^ mice compared with *Thy-Ng2*^*+/+*^; *Braf*^*CA*^ mice (Fig. 5A, B,C). Next, we performed the IHC staining of Ki-67, p-Erk and p-Akt^T308^ in the above tumor sections. The results showed that tumor tissues of *Thy-Ng2*^*−/−*^; *Braf*^*CA*^ mice showed a significant decrease in the number of Ki-67-positive cells and the levels of p-Erk and p-Akt^T308^ upon PLX4720 treatment compared with those of *Thy-Ng2*^*+/+*^; *Braf*^*CA*^ mice (Fig. 5D). The results suggest that NG2 knockout may overcome the feedback activation of ERK and AKT signaling upon BRAF inhibitor treatment. Thus, we treated NG2-knockout 8505 C cells and control cells with 2 µM PLX4720 for different time points. The results showed that NG2 knockout obviously suppressed the rebound of p-ERK and p-AKT^T308^ upon PLX4720 treatment (Fig. 5E), further supporting the above conclusion.

### NG2 knockout decreases the activities of multiple RTKs in thyroid cancer cells upon BRAF inhibitor treatment

**Fig. 6:**
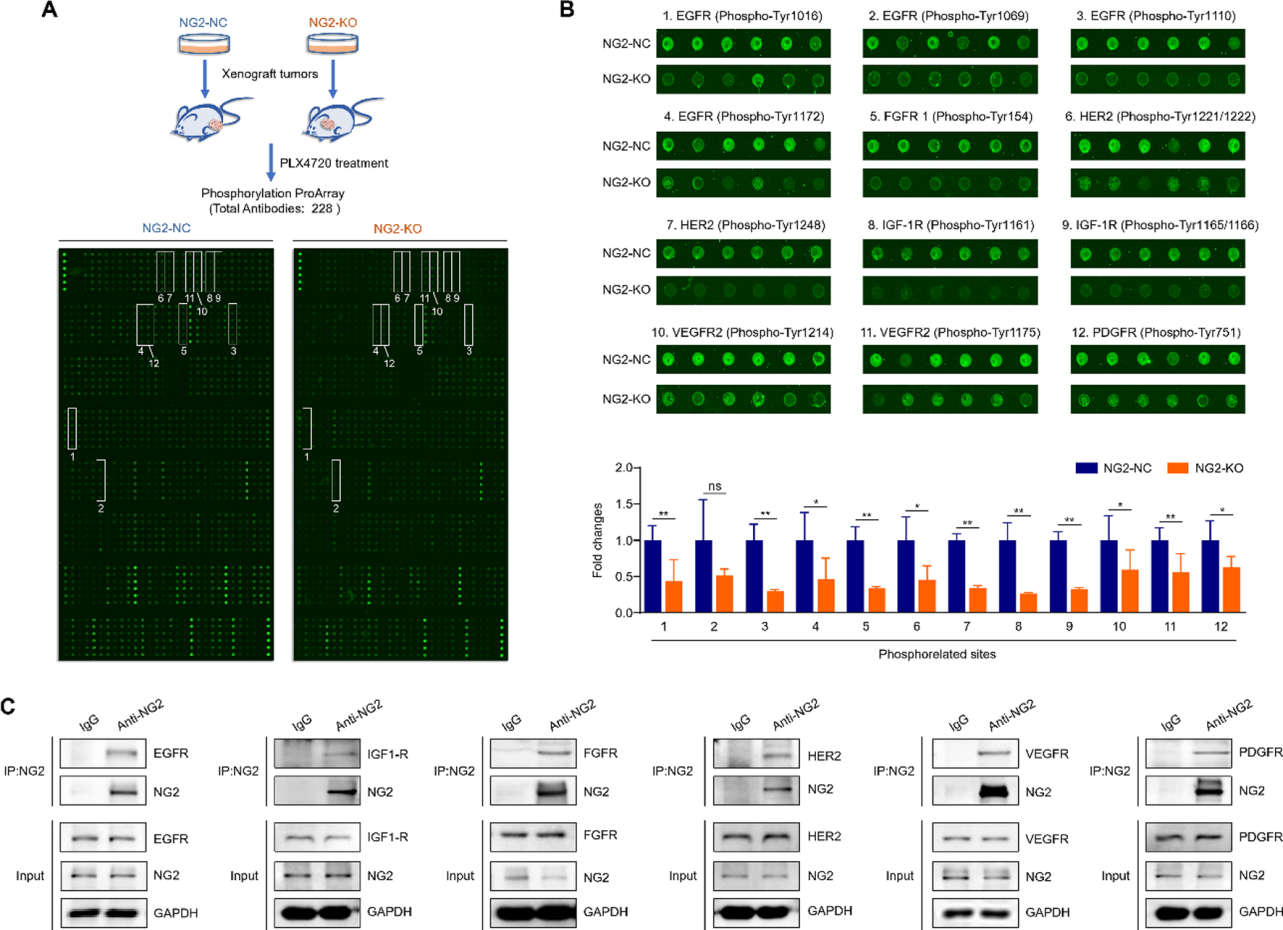
Identification of NG2-regulated RTKs in thyroid cancer cells. (**A**) NG2-knockout 8505 C cell- and control cell-derived xenograft mouse models were administrated daily with PLX4720 (20 mg/kg) were for 5 days. Xenograft tumors were then isolated and homogenized for Tyrosine Phosphorylation ProArray assay. Numbers 1 to 12 indicate an obvious decrease in the levels of tyrosine phosphorylation sites in NG2-knockout tumors (NG2-KO) compared with control tumors (NG2-NC). (**B**) Comparison of the tyrosine phosphorylation levels of the indicated RTKs between NG2-knockout tumors and control tumors upon PLX4720 treatment (upper panels). Lower panel shows the fold changes of the levels of the indicated phosphorylation sites between NG2-knockout tumors and control tumors. (**C**) Co-IP showing the interaction between NG2 and the indicated RTKs, including EGFR, IGF-1R, FGFR, HER2, VEGFR and PDGFR. Data were presented as mean ± SD. *, *P* < 0.05; **, *P* < 0.01; ns, no significance

To determine which RTKs participate in NG2-mediated resistance of BRAF-mutant thyroid cancer cells to BRAF inhibitor, we established NG2-knockout 8505 C cell- and control cell-derived xenograft mouse models, and treated them with PLX4720 (20 mg/kg). After 5 days, mice were sacrificed, xenograft tumors were isolated and homogenized, and the lysates were then analyzed using Tyrosine Phosphorylation ProArray (Wayen Biotechnologies, Inc., Shanghai, China) containing 228 site-specific phospho-tyrosine antibodies (Fig. 6A). The results showed that the tyrosine phosphorylation levels of most RTKs were decreased upon PLX4720 treatment in NG2-knockout tumors compared with control tumors (Supplementary Table 1). Among them, the phosphorylation levels of 12 tyrosine sites of 6 RTKs decreased more significantly than those of other sites, including EGFR (Phospho-Tyr1016, Phospho-Tyr1069, Phospho-Tyr1110 and Phospho-Tyr1172), FGFR (Phospho-Tyr154), HER2 (Phospho-Tyr1221/1222 and Phospho-Tyr1248), IGF-1R (Phospho-Tyr1165/1166), VEGFR2 (Phospho-Tyr1175) and PDGFR (Phospho-Tyr751) (Fig. 6B). In vitro, we also found that ablation of NG2 could down-regulate the phosphorylation of RTKs (Supplementary Fig. 2). In addition, we confirmed the interaction between NG2 and these 6 RTKs by co-immunoprecipitation (co-IP) assay (Fig. 6C).

### An alternative strategy for targeting NG2 to effectively treat BRAF-mutant thyroid cancers by combining BRAF inhibitor with multiple kinase inhibitor (MKI)

**Fig. 7:**
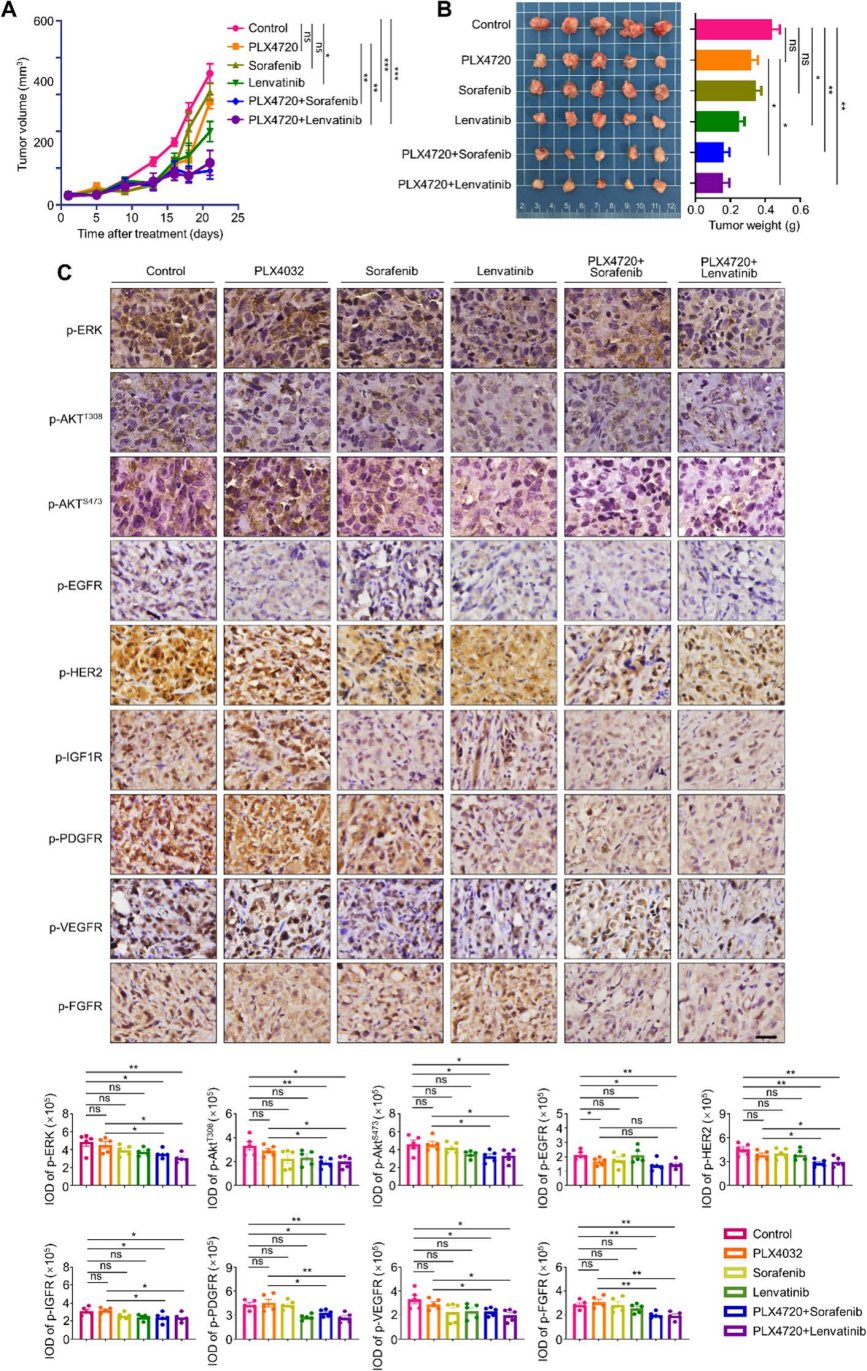
A combination of Sorafenib or Lenvatinib with BRAF inhibitor to treat BRAF-mutant thyroid cancer cells. (**A**) Growth curves of 8505 C cell-derived xenograft tumors with the indicated treatments. (**B**) The photographs of dissected tumors from the indicated groups were shown in the left panel, and statistic results of tumor weight were shown in the right panel. (**C**) IHC staining was performed to evaluate the levels of p-ERK, p-AKT^T308^, p-AKT^S473^, p-EGFR, p-HER2, p-IGF-1R, p-PDGFR, p-VEGFR and p-FGFR. Upper panels show the representative IHC images, and lower panels show the statistic results. Scale bars, 25 μm. IOD, integrated optical density. Data were presented as mean ± SD. *, *P* < 0.05; **, *P* < 0.01; ns, no significance

The above findings indicate that NG2 knockout enhances the sensitivity of BRAF-mutant thyroid cancer cells to BRAF inhibitor. However, there is currently no clinically approved strategy to target NG2 for the treatment of human cancers. Based on the above conclusion, NG2 can sustain the activities of several major RTKs. Some of them happen to be targets of Sorafenib and Lenvatinib, which are already approved by the Food and Drug Administration (FDA) for the treatment of thyroid cancers [16, 17]. Thus, we propose an alternative strategy for targeting NG2 to enhance the efficacy of BRAF inhibitor in BRAF-mutant thyroid cancers. Firstly, the effect of PLX4720, Sorafenib, Lenvatinib and also PLX4720 combined with Sorafenib and Lenvatinib on the proliferation in 8505 C, BCPAP and FTC133 cells were conducted by MTT assay. The result showed that PLX4720 combined with Sorafenib or Lenvatinib significantly inhibited the proliferation than PLX4720 alone in thyroid cancer cell line with BRAF^V600E^ mutation (8505 C and BCPAP), shown in Supplementary Fig. 3. Then we constructed 8505 C cell-derived xenograft mouse model, and then randomly divided these mice into six groups (*n* = 5/group). They were daily administrated with PLX4720 (20 mg/kg), Sorafenib (25 mg/kg), Lenvatinib (25 mg/kg), a combination of PLX4720 and Sorafenib (PLX4720 + Sorafenib), a combination of PLX4720 and Lenvatinib (PLX4720 + Lenvatinib) or the same volume of DMSO for 3 weeks, respectively. During treatment, we recorded the volume of xenograft tumors and plotted their growth curves. As shown in (Fig. 7A), PLX4720 and Sorafenib monotherapy slightly delayed tumor growth, while Lenvatinib monotherapy significantly suppressed tumor growth compared with the control. Inhibitory effect of PLX4720 was further enhanced when combined with Sorafenib or Lenvatinib, especially the latter. Mice were sacrificed after a 3-week of gastric gavage, and xenograft tumors were then isolated and weighted rapidly. The results further supported the above conclusions (Fig. 7B). The liver function tests after the treatment with PLX4720 and combination with Sorafenib and Lenvatinib were also showed in Supplementary Fig. 4.

We next evaluated the levels of p-ERK, p-AKT^T308^, p-AKT^S473^, p-EGFR, p-HER2, p-IGF1R, p-PDGFR, p-VEGFR and p-FGFR in tumor tissues. The results showed that a combination of PLX4720 and Sorafenib or Lenvatinib significantly decreased their levels compared with the control or PLX4720 treatment alone, except for p-EGFR (Fig. 7C). These findings suggest that Sorafenib or Lenvatinib treatment relieves PLX4720-induced feedback activation of RTKs, thereby improving the response of BRAF-mutant thyroid cancer cells to BRAF inhibitor.

## Discussion

The discovery of new biomarkers for thyroid cancer has significantly improved the understanding of the molecular mechanism of thyroid carcinogenesis, thus allowing more personalized treatments for patients with thyroid cancer. However, in the setting of these advances, effective treatments for advanced and metastatic, iodine-refractory thyroid cancer are still limited [18]. Currently, the established treatment approach for differentiated thyroid cancers (DTCs) involves surgical resection as the primary intervention, followed by the administration of radioactive iodine (RAI) therapy [19]. Resistance to repetitive treatment and the development of radioiodine-refractory differentiated thyroid cancers (RAIR-DTCs) are significant contributors to mortality in thyroid cancer patients [20]. Unfortunately, effective treatments for these conditions remain elusive, posing a major challenge in thyroid cancer management.

*BRAF* mutations, especially *BRAF*^*V600E*^ mutation has been identified as a key factor across a wide variety of malignancies, which is also associated with resistance to radioactive iodine therapy [21]. These found have led to the development approval of three BRAF inhibitors as well as five combinations of a BRAF inhibitor to manage cancer such as anaplastic thyroid cancer, melanoma, non-small cell lung cancer and colorectal cancer [18]. Although its inhibitors dabrafenib and vemurafenib have demonstrated potential therapeutic efficacy for BRAF-mutant thyroid cancers [22, 23], their widespread utilization in clinical practice has been limited partly due to the emergence of drug resistance [24]. Thus, there is an urgent need to explore the mechanisms underlying the resistance to BRAF inhibitors in BRAF-mutant thyroid cancers and develop effective strategies to improve their sensitivity to BRAF inhibitors.

Several studies have raised the possible mechanisms of the resistance to BRAF inhibitors in BRAF-mutant thyroid cancers. For example, a previous study has evidenced that the reactivation of ERK signaling as a major effector of *BRAF*^*V600E*^ mutation is strongly associated with compensatory activation of upstream RTKs [25]. For example, BRAF inhibitors promote the transcription of HER3 by releasing the transcription repressor CTBP from its promoter via the inhibition of ERK signaling. Autocrine-secreted NRG1 as a specific ligand of HER3 activates its downstream signaling pathways such as MAPK/ERK and PI3K/AKT pathways by triggering HER3/HER2 heterodimerization and receptor phosphorylation, thereby leading to the resistance to BRAF inhibitors [9]. These observations highlight the role of RTKs and their activation in mediating the resistance of BRAF-mutant thyroid cancer cells to BRAF inhibitors.

In recent years, the role of proteoglycan in tumor has become an intriguing subject [26]. Among the proteoglycans, NG2 is a large cell-surface antigen and an unusual cell membrane integral glycoprotein which was reported to play an important role in tumor cell growth, metastasis and survival as a high-affinity receptor for extracellular proteins, growth factors and integrins [14]. Although the biological significance underlying NG2 proteoglycan involvement in cancer progression needs to be better defined, the expression of NG2 is closely associated with the poor prognoses in hepatocellular carcinoma, pancreatic malignancy and anaplastic thyroid cancer [27–29]. Importantly, NG2-specific monoclonal antibodies (mAbs) have been evidenced to inhibit tumor cell growth and metastasis [30], but have not been approved by the FDA yet. Besides, the latest studies have shown intense preclinical activity of NG2-targeting technology in both melanoma and breast cancer, further supporting NG2 as a valuable target in cancer therapy [29, 31].

In the present study, we find that NG2 was up-regulated in thyroid cancers compared with control subjects; however, knocking out NG2 in thyroid cancer cells almost did not change their proliferation and colony formation abilities, as supported by the results of xenograft mouse model and genetically engineered mouse model. Surprisedly, we found that NG2 knockout improved the response of BRAF-mutant thyroid cancer cells to BRAF inhibitor using the above animal models. Besides, our data also demonstrated that NG2 knockout attenuated the rebound of p-ERK and p-AKT^T308^ upon BRAF inhibitor treatment in BRAF-mutant thyroid cancer cells. These findings highlight that targeting NG2 may be a potential strategy to overcome the resistance of BRAF-mutant thyroid cancer cells to BRAF inhibitors.

Further studies showed that NG2 was involved in maintaining the activities of multiple RTKs, such as EGFR, FGFR, HER2, IGF-1R, VEGFR and PDGFR, suggesting that these RTKs and their downstream signaling pathways may contribute to the resistance of BRAF-mutant thyroid cancer cells to BRAF inhibitor. Recent advancements in emerging targeted drugs have expanded the treatment options for thyroid cancer management. Among them, Sorafenib, an MKI, has been approved by the FDA for the treatment of advanced thyroid cancers that are unresponsive to radioactive iodine therapy. Studies have demonstrated that Sorafenib substantially enhances the progression-free survival in DTC patients who exhibit resistance to radioactive iodine therapy compared with the placebo [32]. Importantly, the adverse reactions associated with Sorafenib treatment have been evidenced as manageable [32]. Lenvatinib is another MKI approved by FDA for the treatment of DTC patients. Studies have shown that Lenvatinib significantly extends the progression-free survival (PFS) compared with the placebo in RAIR-DTC patients [33, 34]. Sorafenib and Lenvatinib are broad-spectrum inhibitors of host receptor tyrosine kinases (RTKs). Fortunately, some of NG2-regulated RTKs are the potential targets of Sorafenib or Lenvatinib, suggesting that mechanism of action of Sorafenib or Lenvatinib partially overlaps with NG2 knockout.

According to these observations, we propose an alternative strategy for targeting NG2 to treat BRAF-mutant thyroid cancers by combining Sorafenib or Lenvatinib with BRAF inhibitor. Expectedly, our data showed that Sorafenib or Lenvatinib treatment effectively improved the anti-tumor efficacy of BRAF inhibitor PLX4720, and relieved the feedback activation of PLX4720-mediated RTKs and their downstream effectors ERK and AKT cascades. Thus, this is a promising therapeutic strategy for BRAF-mutant thyroid cancers. However, the present study still has some limitations. For example, this study demonstrates the potential role of NG2 in alleviating BRAF inhibitor resistance by cell line, NG2-knockout xenograft tumor model and thyroid-specific NG2 knockout mouse model. Similar NG2-targeting models, such as CAR-T and monoclonal antibody models, should be used for further exploration which will be closer to clinical application.

**Fig. 8:**
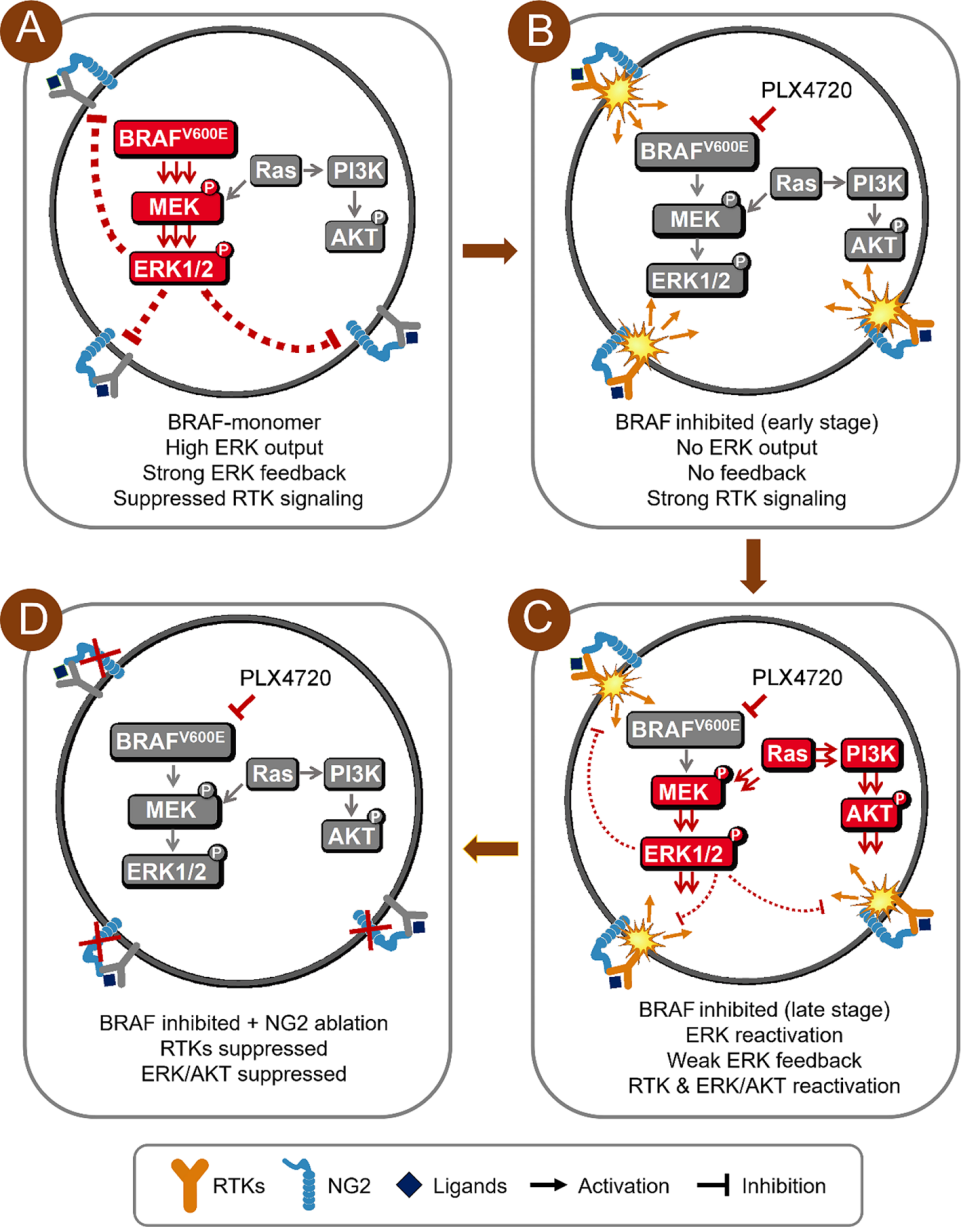
A schematic model of NG2 relieving the resistance of BRAF-mutant thyroid cancer cells to BRAF inhibitor. (**A**) ERK signaling is overactivated in BRAF-mutant thyroid cancer cells, and high levels of p-ERK lead to a strong feedback inhibition of RTK signaling. (**B**) The early stage of BRAF inhibitor treatment, ERK signaling is blocked, leading to the reactivation of RTK signaling. (**C**) RTKs and their downstream cascades such as MAPK/ERK and PI3K/AKT pathways are reactivated, although there is a weak ERK feedback at the late stage of BRAF inhibitor treatment. (**D**) A combined treatment of BRAF inhibitor and NG2 ablation significantly relieves the feedback activation of RTKs and their downstream signaling pathways, thereby improving the response of BRAF-mutant thyroid cancer cells to BRAF inhibitor

In summary, we find that NG2 is up-regulated in thyroid cancers and associated with tumor progression. Moreover, we demonstrate that NG2 knockout does not affect tumor growth in vitro and in vivo, but enhances the response of BRAF-mutant thyroid cancer cells to BRAF inhibitor. The corresponding mechanism is elucidated by a simple model (Fig. 8). In general, the output of p-ERK is relatively high in BRAF-mutant thyroid cancer cells. High levels of p-ERK cause a strong feedback inhibition of RTK signaling. Blockade of ERK signaling by BRAF inhibitor inactivates ERK-dependent feedback, and then reactivates multiple RTKs and their downstream ERK and AKT signaling cascades, thereby causing the resistance of BRAF-mutant thyroid cancer cells to BRAF inhibitor. In this process, NG2 acts as an important player in reactivating RTK signaling, as supported by our data showing that NG2 knockout relieves the feedback activation of RTKs and improves the response of BRAF-mutant thyroid cancer cells to BRAF inhibitor. Based on the above findings, this study raises an alternative strategy for targeting NG2 to effectively treat BRAF-mutant thyroid cancer cells by combining Sorafenib or Lenvatinib and BRAF inhibitor.

## Materials and methods

### Cell culture and drug treatment

Human thyroid cancer cell lines 8505 C (with BRAF^V600E^ mutation), BCPAP (with BRAF^V600E^ mutation) and FTC133(without BRAF^V600E^ mutation) were kindly provided by Dr. Haixia Guan (Department of Endocrinology, Guangdong Provincial People’s Hospital, China). Prior to their use, all cell lines underwent authentication through short tandem repeat (STR) analysis conducted at Genesky Co. Ltd (Shanghai, China). The STR analysis results were further compared with a previous study [35] and the COSMIC database (https://cancer.sanger.ac.uk/cell_lines), ensuring the consistency and reliability of the cell line identities. These cell lines were routinely cultured in RPMI 1640 or DMEM medium with 10% fetal bovine serum (FBS). In some experiments, thyroid cancer cells were treated with 2µM BRAF-specific inhibitor PLX4720 (MCE, #HY-51,424) and dissolved in dimethyl sulfoxide (DMSO). The same volume of DMSO was used as control.

### Clinical samples

With the institutional review board approval and patient consent, a total of 20 PTC tissues (with 85% of the patients acquired a BRAF^V600E^ mutation, *n* = 17) and their paired non-cancerous thyroid tissues (control subjects) were obtained from the First Affiliated Hospital of Xi’an Jiaotong University. All patients did not receive radioiodine treatment or other anti-tumor medications prior to surgery, and all tissues were histologically examined by a senior pathologist at the Department of Pathology of the Hospital based on World Health Organization (WHO) criteria.

### Animal studies

All animal studies were approved by the Institutional Review Board of Xi’an Jiaotong University Health Science Center (Approval No. XJTU2019-G195). The transgenic mouse strains *Ng2*^*flox/flox*^ C57Bl/6 used in the study were kindly provided by Prof. William Stallcup (Sanford Burnham Prebys Medical Discovery Institute, USA). The *Tpo-Cre* and *LSL-Braf*^*CA*^ mouse strains were kindly provided by Prof. Kimura Shioko (the National Institutes of Health, USA). *Ng2*^*flox/flox*^ mice, *LSL-Braf*^*CA*^ mice and *Tpo-Cre* mice were crossed to obtain thyroid-specific Ng2 knockout coupled with *Braf*^*V600E*^-driven thyroid cancer mouse model (*Thy-Ng2*^*−/−*^; *Braf*^*CA*^). Their wild-type littermates were used as controls (*Thy-Ng2*^*+/+*^; *Braf*^*CA*^). The above mouse lines were genotyped by PCR using tail DNA (Supplementary Fig. 5), and the sequences of specific primers were provided in Supplementary Table 2.

To determine the effect of NG2 knockout on tumor growth in nude mice, female nude mice aged 5–6 weeks were purchased from SLAC Laboratory Animal Co., Ltd. (Shanghai, China) and randomly divided into two groups (*n* = 6/group). 8505 C (with BRAF ^V600E^ mutation and relatively high NG2 expression) were used for the in vivo study. Next, NG2-knockout 8505 C cells (NG2-KO, 6 × 10^ × 6^ cells) or control cells (NG2-NC) were injected subcutaneously into the flanks of nude mice to construct xenograft tumor models. Tumor sizes were measured every other day, and tumor volumes were then calculated using the formula (length × width^2^ × 0.5). To determine the effect of NG2 knockout on the anti-tumor efficacy of PLX4720, we similarly established xenograft tumor models using NG2-knockout 8505 C cells and control cells, and divided them into 4 groups (*n* = 5/group): NG2-NC + Control, NG2-NC + PLX4720, NG2-KO + Control and NG2-KO + PLX4720. To explore PLX4720 and its synergies with other method while minimizing toxic side effects, a lower dose of PLX4720 was adopted (20 mg/kg/day) for a 2-week treatment [36]. When tumor volumes reached 10–50 mm^3^, the mice were treated orally with BRAF inhibitor PLX4720 at a dose of 20 mg/kg/day or the vehicle (DMSO) for a duration of 2 weeks. Tumor volume and weight were then measured using the methods described above.

To determine the anti-tumor efficacy of Sorafenib or Lenvatinib in combination with BRAF inhibitor, we constructed the 8505 C cell- and control cell-derived xenograft mouse models, and divided them into 6 groups (*n* = 5/group): Control, PLX4720, Sorafenib, Lenvatinib, PLX4720 + Sorafenib, and PLX4720 + Lenvatinib. When the tumors reached the volumes as mentioned above, the mice were treated orally with DMSO, PLX4720 at a dose of 20 mg/kg/day, Sorafenib (Selleck Chemicals, #S1040) at a dose of 25 mg/kg/day and Lenvatinib (Selleck Chemicals, # S1164) at a dose of 25 mg/kg/day, individually or in combination, for three weeks. We measured the tumor volumes and weight based on the similar methods. At the end of all experiments, all mice were sacrificed and tumors were then isolated and weighed.

### RNA extraction and quantitative RT-PCR (qRT-PCR)

RNA extraction, cDNA preparation and qRT-PCR were carried out as described previously [37]. The primer sequences were presented in Supplementary Table 3. Each sample was run in triplicate, and 18 S rRNA was used as a reference gene to normalize the mRNA expression of Ng2.

### Western blotting and co-immunoprecipitation (co-IP) assays

Thyroid cancer cells were cultured until they reached approximately 80% confluence. After washing with PBS, cells were mechanically lysed using RIPA buffer supplemented with protease inhibitors, allowing for a 30-min incubation. Western blotting and co-immunoprecipitation (co-IP) assays were conducted according to established protocols [15]. The antibodies used in this study were presented in Supplementary Table 4.

### Hematoxylin and Eosin (H&E) staining, immunohistochemistry (IHC) staining, and immunofluorescence (IF) staining

The paraffin sections with a thickness of 5 μm were prepared for H&E staining and IHC staining. IF staining was performed on frozen sections with a thickness of 6 μm. The staining procedures followed previously established protocols [38]. The antibodies used in this study were also presented in Supplementary Table 4. Protein expression levels were quantified by measuring the integral optical density (IOD) using Image-Pro Plus 6.0 software (Media Cybernetics, USA).

### Cell viability and colony formation assays

Thyroid cancer cells were seeded in 96-well plates, and the MTT assays were performed daily for a period of 5 days to evaluate their cell viability according to established protocol [35]. Approximately 3000 to 4000 cells were seeded per well in 6-well plates and cultured in RPMI-1640 or DMEM medium supplemented with 10% FBS for a duration of 7–10 days. The colonies formed were fixed with 4% methanol for 10 min, washed with PBS, and stained with crystal violet for 8 min. Each assay was done in triplicate to ensure reproducibility.

### Lentivirus transfection

NG2 was knocked out in 8505 C, BCPAP and FTC133 cells using the CRISPR/Cas9 system as described previously [39].

### Statistical analysis

The investigator responsible for data analysis were blinded to which samples/animals represent treatments and controls. Statistical analyses were conducted using GraphPad Prism 8.4 software (GraphPad Software, Inc.). The differences between groups were evaluated using the unpaired Student’s t-test or one-way analysis, as appropriate. The data were presented as mean values ± standard deviation (SD). A P-value less than 0.05 (*P* < 0.05) was considered statistically significant.

### Electronic supplementary material

Below is the link to the electronic supplementary material.

Supplementary Material 1

Supplementary Material 2

## Data Availability

All data generated or analyzed during this study are included in this published article and the supplementary files.

## Declarations

### Competing of interest

We declare that the article we submitted on Drug resistance updates has not been published previously, that it is not under consideration for publication elsewhere, that its publication is approved by all authors and tacitly or explicitly by the responsible authorities where the work was carried out, and that, if accepted, it will not be published elsewhere.
