# Supplementary material for: Targeting NG2 relieves the resistance of BRAF-mutant thyroid cancer cells to BRAF inhibitors
Source: Cell Mol Life Sci. 2024 May 25;81(1):238. doi: 10.1007/s00018-024-05280-6 (PMC11127897; doi:10.1007/s00018-024-05280-6)
Supplement: Supplementary file 2 — Supplementary Material 2 [file 18_2024_5280_MOESM2_ESM.docx]

Supplementary Materials for

**Targeting NG2 relieves the resistance of BRAF-mutant thyroid cancer cells to BRAF inhibitors**

Fang Sui^1,2,#^, Guanjie Wang^1,#^, Juan Liu^1^, Mengmeng Yuan^1^, Pu Chen^1^, Yao Yao^1^, Shaoqiang Zhang^2^, Meiju Ji^3,*^, and Peng Hou^1,4,*^

*** Corresponding authors:**

*E-mail addresses*: [phou@xjtu.edu.cn](mailto:phou@xjtu.edu.cn) (Peng Hou), [mjji0409@163.com](mailto:mjji0409@163.com) (Meiju Ji).

**This file includes:**

Tables S1 to S4; Table S1 is a separate excel file.

Figures S1 -S5


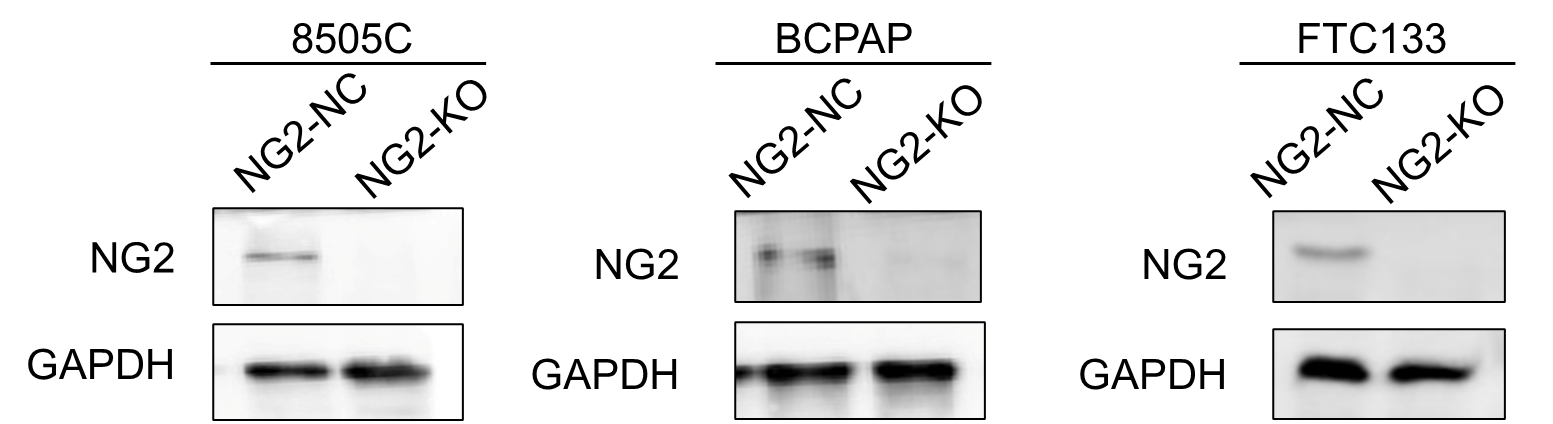


**Fig. S1.** NG2 knockout efficiency in 8505C BCPAP and FTC133.

NG2 was knocked out in 8505C, BCPAP and FTC133 cells using the CRISPR/Cas9 system. Western blot assay was used for further validation. GAPDH was used as a loading control.


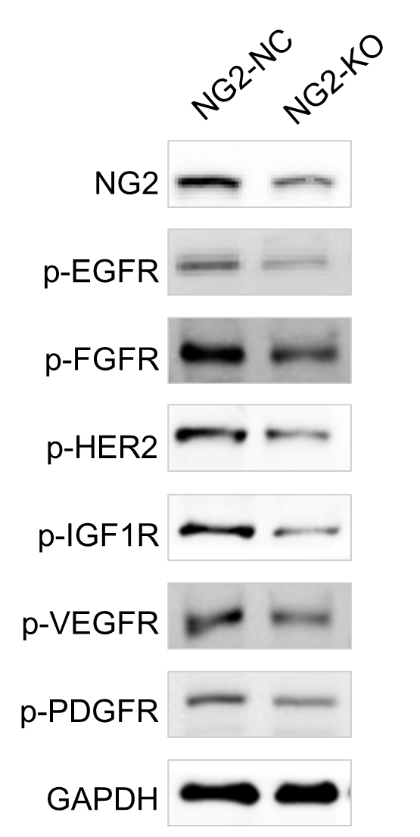


**Fig. S2.** The effect of NG2 ablation on phosphorylated RTKs in vitro.

NG2 was knocked out in 8505C cells using the CRISPR/Cas9 system. Western blot assay was performed to assess the expression of p-EGFR, p-FGFR, p-HER2, p-IGF-1R, p-VEGFR and p-PDGFR. GAPDH was used as a loading control.


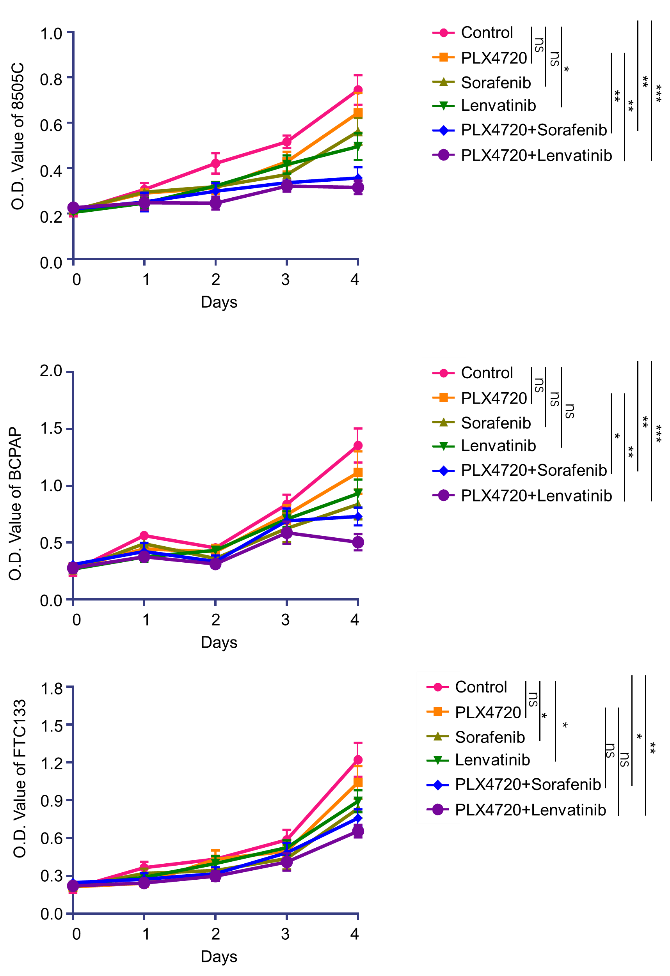


**Fig. S3.** The effect of PLX4720, Sorafenib and Lenvatinib on the proliferation of thyroid cancer.

MTT assay were conducted to assess the effect of PLX4720 (1μM), Sorafenib (1μM), Lenvatinib (1μM) and also PLX4720 (1μM) combined with Sorafenib (1μM) and Lenvatinib (1μM) on the proliferation in 8505C, BCPAP and FTC133 cells.


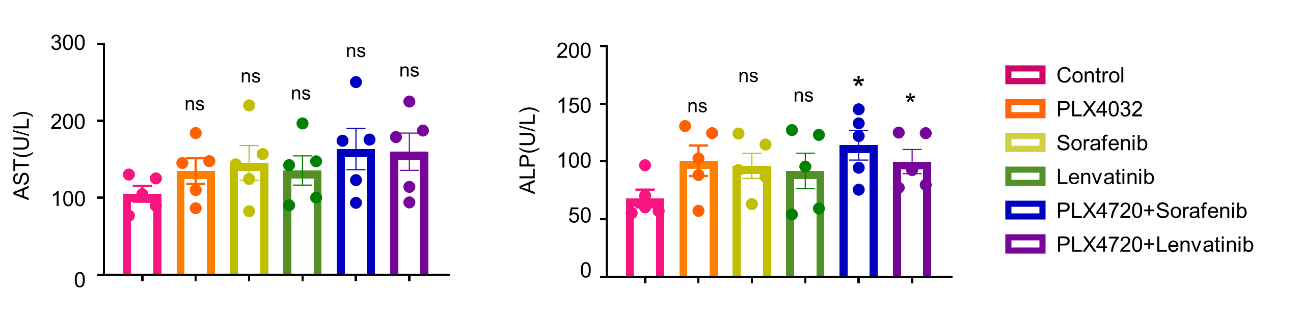


**Fig. S4.** The AST and ALP of mice after indicated treatment.

In the 8505C cell-derived xenograft mouse model (n =5/group). The mice were daily administrated with PLX4720 (20 mg/kg), Sorafenib (25 mg/kg), Lenvatinib (25 mg/kg), a combination of PLX4720 and Sorafenib (PLX4720+Sorafenib), a combination of PLX4720 and Lenvatinib (PLX4720+ Lenvatinib) or the same volume of DMSO for 3 weeks, respectively. The Mice were sacrificed and the AST and ALP of the mice was assessed. AST, Aspartate aminotransferase; ALP, Alkaline phosphatase.


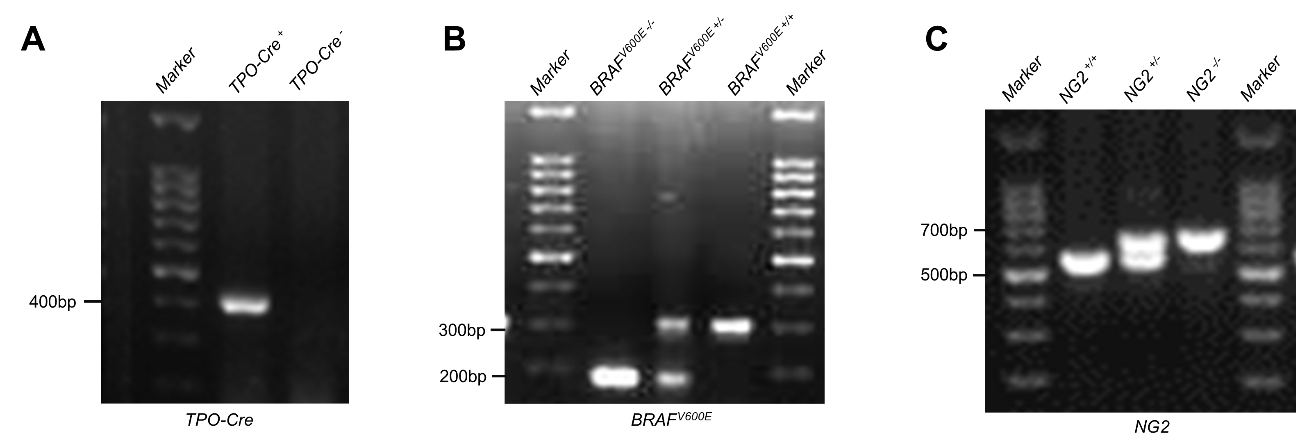


**Fig. S5.** Genotyping of genetically engineered mice.

The above mouse lines were genotyped by PCR using tail DNA, and the sequences of specific primers were provided in Supplementary Table 2.

**Supplementary Tables**

**Table S2**. The primers used for genotyping

| **Genes** | **Sequences (5′-3′)** |
| --- | --- |
| *Ng2* (sense) | TGCTCCAGTTCTCCACAT |
| *Ng2* (antisense) | CTATCTCAGATTGTACCACCTT |
| *Tpo-Cre* (sense) | AGGTGTAGAGAAGGCACTTAGC |
| *Tpo-Cre* (antisense) | CTAATCGCCATCTTCCAGCAGG |
| *Braf^CA^* (sense) | TGAGTATTTTTGTGGCAACTGC |
| *Braf^CA^* (antisense) | CTCTGCTGGGAAAGCGGC |

**Table S3**. The primers used for qRT-PCR

| **Genes** | **Sequences (5′-3′)** |
| --- | --- |
| Mouse - *Ng2* (sense) | GAGGTCTTGGTGAACTTCACCC |
| Mouse - *Ng2* (antisense) | GACAGTAGGAGACCGATGGTGT |
| Mouse - *18S* (sense) | CGCCGCTAGAGGTGAAATTC |
| Mouse - *18S* (antisense) | CTTTCGCTCTGGTCCGTCTT |

**Table S4**. The antibodies used in this study

| **Antibodies** | **Source** |
| --- | --- |
| Anti-NG2 | Abcam |
| Anti-GAPDH | Abgent |
| Anti- Ki67 | BD Pharmingen |
| Anti- AKT (phospho-308) | Bioworld Technology |
| Anti- AKT (phospho-473) | Bioworld Technology |
| Anti - total - AKT | Bioworld Technology |
| Anti – total - ERK | Cell signaling Technology |
| Anti - phospho ERK1/2 | Cell signaling Technology |
| Anti – HER2 | Santa Cruz |
| Anti- HER2 (Phospho-Tyr1221/1222) | Cell signaling Technology |
| Anti- HER2(Phospho-Tyr1248) | Cell signaling Technology |
| Anti- EGFR | Cell signaling Technology |
| Anti- EGFR (Phospho-Tyr1016) | Affinity Biosciences |
| Anti- EGFR (Phospho-Tyr1110) | Affinity Biosciences |
| Anti- IGF1R | Cell signaling Technology |
| Anti-IGF-1R (Phospho-Tyr1165/1166) | Cell signaling Technology |
| Anti- IGF-1R (Phospho-Tyr1161) | Abcam |
| Anti- FGFR | Abcam |
| Anti- FGFR1 (Phospho-Tyr154) | Cell signaling Technology |
| Anti-VEGFR | Cell signaling Technology |
| Anti-VEGFR (Phospho-Tyr1175) | Cell signaling Technology |
| Anti-PDGF-R | Cell signaling Technology |
| Anti-PDGF-R beta (Phospho-Tyr751) | Cell signaling Technology |
